# *N*,*N*′-Bis(2,3-dimeth­oxy­benzyl­idene)ethane-1,2-diamine

**DOI:** 10.1107/S1600536811033423

**Published:** 2011-08-27

**Authors:** Hua Xue, Wenjuan Li, Hongfei Han

**Affiliations:** a Institute of Applied Chemistry, Taiyuan Normal University, Taiyuan 030031, People’s Republic of China; b Department of Chemistry, Taiyuan Normal University, Taiyuan 030031, People’s Republic of China

## Abstract

The title compound, C_20_H_24_N_2_O_4_, crystallizes with two half (centrosymmetric) mol­ecules in the asymmetric unit. There are only minor differences between the geometric parameters between these two mol­ecules. The two aromatic rings in both mol­ecules are mutually coplanar.

## Related literature

For general background to the properties of Schiff bases, see: Layer (1963 ▶); Chen *et al.* (2008 ▶); May *et al.* (2004 ▶). For related structures, see: Harada *et al.* (2004 ▶); Tariq *et al.* (2010 ▶).
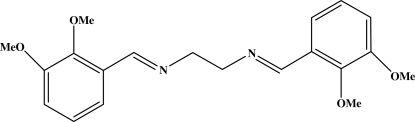


## Experimental

### 

#### Crystal data

C_20_H_24_N_2_O_4_
*M* *_r_* = 356.41
Triclinic, 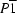

*a* = 5.0491 (5) Å
*b* = 13.5803 (15) Å
*c* = 13.5803 (15) Å
α = 89.866 (2)°
β = 88.863 (1)°
γ = 88.863 (1)°
*V* = 930.81 (17) Å^3^
*Z* = 2
Mo *K*α radiation
μ = 0.09 mm^−1^
*T* = 298 K
0.47 × 0.41 × 0.40 mm

#### Data collection

Bruker SMART CCD area-detector diffractometer
Absorption correction: multi-scan (*SADABS*; Sheldrick, 1996 ▶) *T*_min_ = 0.959, *T*_max_ = 0.965
4767 measured reflections
3207 independent reflections
1811 reflections with *I* > 2σ(*I*)
*R*_int_ = 0.034

#### Refinement

*R*[*F* ^2^ > 2σ(*F* ^2^)] = 0.062
*wR*(*F* ^2^) = 0.184
*S* = 1.04
3207 reflections
239 parameters
H-atom parameters constrained
Δρ_max_ = 0.23 e Å^−3^
Δρ_min_ = −0.26 e Å^−3^

### 

Data collection: *SMART* (Bruker, 2007 ▶); cell refinement: *SAINT* (Bruker, 2007 ▶); data reduction: *SAINT*; program(s) used to solve structure: *SHELXS97* (Sheldrick, 2008 ▶); program(s) used to refine structure: *SHELXL97* (Sheldrick, 2008 ▶); molecular graphics: *SHELXTL* (Sheldrick, 2008 ▶); software used to prepare material for publication: *SHELXTL*.

## Supplementary Material

Crystal structure: contains datablock(s) I, global. DOI: 10.1107/S1600536811033423/bt5615sup1.cif

Structure factors: contains datablock(s) I. DOI: 10.1107/S1600536811033423/bt5615Isup2.hkl

Supplementary material file. DOI: 10.1107/S1600536811033423/bt5615Isup3.cml

Additional supplementary materials:  crystallographic information; 3D view; checkCIF report

## Figures and Tables

**Fig. 1.**
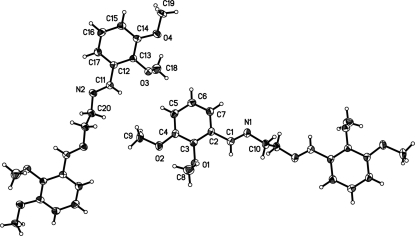
The molecular structure of the title compound, showing the atom–numbering scheme. Displacement ellipsoids are drawn at the 30% probability level.

## supplementary crystallographic information

### Comment

Schiff bases containing the C═N bond have been receiving considerable attention for many years, primarily due to their importance as ligands in metal complexes with special biological (May *et al.*, 2004), and catalytic properties (Chen *et al.*, 2008).

As a part of our studies on synthesis and structural peculiarities of Schiff bases derived from 1,2-diaminoethane and 2,3-dimethoxybenzaldehyde, we determined the structure of the title compound (Fig. 1). The molecule includes two C═N bonds, which are coplanar. The distance between the C atom and the N atom in the C═N bond is 1.254 (4) Å. The two benzene rings in the structure are parallel each other.

### Experimental

1,2-Diaminoethane (0.180 g, 3 mmol) was added dropwise with stirring at 0°C to a solution of 2,3-dimethoxybenzaldehyde (0.997 g, 6 mmol) in ethanol. The mixture was warmed to room temperature and stirred for 2 h. The reaction mixture was filtered and the filter cake was recrystallized from ethanol (yield 80%). Crystals of the title compound suitable for X-ray diffraction were obtained by slow evaporation of a tetrahydrofuran solution.

### Refinement

All H atoms were positioned geometrically(C—H = 0.93–0.98 Å), and refined as riding with *U*_iso_(H) = 1.2*U*_eq_ of the adjacent carbon atom (1.5*U*_eq_ for methyl H atoms).

### Crystal data

**Table:**

C_20_H_24_N_2_O_4_	*Z* = 2
*M**_r_* = 356.41	*F*(000) = 380
Triclinic, *P*1	*D*_x_ = 1.272 Mg m^−^^3^
*a* = 5.0491 (5) Å	Mo *K*α radiation, λ = 0.71073 Å
*b* = 13.5803 (15) Å	Cell parameters from 1477 reflections
*c* = 13.5803 (15) Å	θ = 3.0–25.3°
α = 89.866 (2)°	µ = 0.09 mm^−^^1^
β = 88.863 (1)°	*T* = 298 K
γ = 88.863 (1)°	Block, colorless
*V* = 930.81 (17) Å^3^	0.47 × 0.41 × 0.40 mm

### Data collection

**Table:**

Bruker SMART CCD area-detector diffractometer	3207 independent reflections
Radiation source: fine-focus sealed tube	1811 reflections with *I* > 2σ(*I*)
graphite	*R*_int_ = 0.034
φ and ω scans	θ_max_ = 25.0°, θ_min_ = 3.0°
Absorption correction: multi-scan (*SADABS*; Sheldrick, 1996)	*h* = −5→6
*T*_min_ = 0.959, *T*_max_ = 0.965	*k* = −11→16
4767 measured reflections	*l* = −16→15

### Refinement

**Table:**

Refinement on *F*^2^	Primary atom site location: structure-invariant direct methods
Least-squares matrix: full	Secondary atom site location: difference Fourier map
*R*[*F*^2^ > 2σ(*F*^2^)] = 0.062	Hydrogen site location: inferred from neighbouring sites
*wR*(*F*^2^) = 0.184	H-atom parameters constrained
*S* = 1.04	*w* = 1/[σ^2^(*F*_o_^2^) + (0.087*P*)^2^ + 0.1177*P*] where *P* = (*F*_o_^2^ + 2*F*_c_^2^)/3
3207 reflections	(Δ/σ)_max_ < 0.001
239 parameters	Δρ_max_ = 0.23 e Å^−^^3^
0 restraints	Δρ_min_ = −0.26 e Å^−^^3^

### Special details

**Table:**

Geometry. All e.s.d.'s (except the e.s.d. in the dihedral angle between two l.s. planes) are estimated using the full covariance matrix. The cell e.s.d.'s are taken into account individually in the estimation of e.s.d.'s in distances, angles and torsion angles; correlations between e.s.d.'s in cell parameters are only used when they are defined by crystal symmetry. An approximate (isotropic) treatment of cell e.s.d.'s is used for estimating e.s.d.'s involving l.s. planes.
Refinement. Refinement of *F*^2^ against ALL reflections. The weighted *R*-factor *wR* and goodness of fit *S* are based on *F*^2^, conventional *R*-factors *R* are based on *F*, with *F* set to zero for negative *F*^2^. The threshold expression of *F*^2^ > σ(*F*^2^) is used only for calculating *R*-factors(gt) *etc*. and is not relevant to the choice of reflections for refinement. *R*-factors based on *F*^2^ are statistically about twice as large as those based on *F*, and *R*- factors based on ALL data will be even larger.

### Fractional atomic coordinates and isotropic or equivalent isotropic displacement parameters (Å^2^)

**Table:**

	*x*	*y*	*z*	*U*_iso_*/*U*_eq_	
N1	0.8830 (5)	0.89133 (19)	0.93288 (19)	0.0473 (7)	
N2	−0.3832 (5)	0.39082 (19)	0.56712 (18)	0.0465 (7)	
O1	0.3838 (4)	0.91237 (15)	0.70797 (15)	0.0485 (6)	
O2	0.0726 (4)	0.76450 (16)	0.65165 (16)	0.0531 (6)	
O3	0.1160 (4)	0.41215 (15)	0.79204 (15)	0.0485 (6)	
O4	0.4260 (4)	0.26460 (16)	0.84843 (16)	0.0530 (6)	
C1	0.7533 (6)	0.8916 (2)	0.8551 (2)	0.0412 (8)	
H1	0.7668	0.9451	0.8126	0.049*	
C2	0.5804 (6)	0.8102 (2)	0.8286 (2)	0.0382 (7)	
C3	0.4037 (6)	0.8220 (2)	0.7529 (2)	0.0376 (7)	
C4	0.2413 (6)	0.7449 (2)	0.7263 (2)	0.0403 (8)	
C5	0.2564 (7)	0.6562 (2)	0.7739 (2)	0.0476 (9)	
H5	0.1496	0.6046	0.7557	0.057*	
C6	0.4331 (7)	0.6448 (2)	0.8495 (2)	0.0536 (9)	
H6	0.4449	0.5848	0.8825	0.064*	
C7	0.5912 (7)	0.7204 (2)	0.8764 (2)	0.0486 (9)	
H7	0.7080	0.7111	0.9279	0.058*	
C8	0.5069 (9)	0.9172 (3)	0.6126 (3)	0.0718 (12)	
H8A	0.6926	0.9019	0.6176	0.108*	
H8B	0.4839	0.9824	0.5862	0.108*	
H8C	0.4270	0.8706	0.5698	0.108*	
C9	−0.0886 (7)	0.6865 (3)	0.6191 (3)	0.0581 (10)	
H9A	0.0225	0.6333	0.5948	0.087*	
H9B	−0.2003	0.7100	0.5673	0.087*	
H9C	−0.1965	0.6636	0.6731	0.087*	
C10	1.0447 (6)	0.9765 (2)	0.9517 (2)	0.0475 (8)	
H10A	1.0266	1.0238	0.8985	0.057*	
H10B	1.2296	0.9563	0.9550	0.057*	
C11	−0.2531 (6)	0.3919 (2)	0.6447 (2)	0.0401 (8)	
H11	−0.2679	0.4464	0.6860	0.048*	
C12	−0.0791 (6)	0.3099 (2)	0.6716 (2)	0.0371 (7)	
C13	0.0954 (6)	0.3223 (2)	0.7471 (2)	0.0366 (7)	
C14	0.2596 (6)	0.2440 (2)	0.7735 (2)	0.0410 (8)	
C15	0.2442 (7)	0.1563 (2)	0.7261 (2)	0.0476 (8)	
H15	0.3533	0.1038	0.7447	0.057*	
C16	0.0680 (7)	0.1443 (2)	0.6506 (2)	0.0542 (9)	
H16	0.0591	0.0840	0.6187	0.065*	
C17	−0.0920 (7)	0.2208 (2)	0.6233 (2)	0.0478 (8)	
H17	−0.2097	0.2131	0.5722	0.057*	
C18	−0.0072 (9)	0.4175 (3)	0.8874 (3)	0.0732 (12)	
H18A	−0.1927	0.4047	0.8823	0.110*	
H18B	0.0152	0.4820	0.9143	0.110*	
H18C	0.0731	0.3693	0.9298	0.110*	
C19	0.5896 (7)	0.1861 (3)	0.8810 (3)	0.0576 (10)	
H19A	0.4812	0.1339	0.9058	0.086*	
H19B	0.7008	0.2088	0.9324	0.086*	
H19C	0.6979	0.1621	0.8270	0.086*	
C20	−0.5456 (6)	0.4766 (2)	0.5481 (2)	0.0466 (8)	
H20A	−0.5313	0.5233	0.6015	0.056*	
H20B	−0.7296	0.4581	0.5437	0.056*	

### Atomic displacement parameters (Å^2^)

**Table:**

	*U*^11^	*U*^22^	*U*^33^	*U*^12^	*U*^13^	*U*^23^
N1	0.0444 (17)	0.0526 (17)	0.0455 (16)	−0.0109 (13)	−0.0038 (13)	−0.0035 (12)
N2	0.0435 (17)	0.0508 (17)	0.0450 (16)	0.0084 (13)	−0.0041 (13)	0.0012 (12)
O1	0.0537 (15)	0.0412 (13)	0.0506 (14)	0.0013 (10)	−0.0041 (11)	0.0001 (10)
O2	0.0421 (14)	0.0577 (15)	0.0600 (14)	−0.0042 (11)	−0.0126 (11)	−0.0077 (11)
O3	0.0510 (14)	0.0437 (13)	0.0511 (14)	−0.0042 (11)	−0.0052 (11)	0.0006 (10)
O4	0.0422 (14)	0.0563 (15)	0.0609 (14)	0.0030 (11)	−0.0122 (11)	0.0088 (11)
C1	0.0410 (19)	0.0426 (18)	0.0399 (18)	−0.0035 (15)	0.0031 (15)	−0.0032 (14)
C2	0.0334 (18)	0.0415 (18)	0.0396 (17)	−0.0047 (14)	0.0039 (14)	−0.0055 (14)
C3	0.0364 (18)	0.0353 (18)	0.0410 (17)	−0.0004 (14)	0.0047 (14)	−0.0043 (13)
C4	0.0312 (18)	0.0459 (19)	0.0435 (18)	0.0001 (15)	0.0026 (15)	−0.0100 (14)
C5	0.039 (2)	0.044 (2)	0.060 (2)	−0.0077 (15)	0.0030 (17)	−0.0073 (16)
C6	0.057 (2)	0.046 (2)	0.059 (2)	−0.0063 (17)	−0.0039 (18)	0.0084 (16)
C7	0.049 (2)	0.052 (2)	0.0447 (19)	−0.0042 (17)	−0.0054 (16)	0.0007 (15)
C8	0.092 (3)	0.066 (3)	0.058 (2)	0.000 (2)	0.003 (2)	0.0093 (19)
C9	0.037 (2)	0.070 (2)	0.068 (2)	−0.0042 (18)	−0.0082 (18)	−0.0223 (19)
C10	0.0351 (19)	0.058 (2)	0.0502 (19)	−0.0087 (16)	−0.0039 (15)	0.0004 (15)
C11	0.0389 (19)	0.0429 (18)	0.0385 (18)	0.0000 (15)	0.0017 (15)	0.0005 (13)
C12	0.0324 (17)	0.0377 (18)	0.0410 (17)	0.0000 (14)	0.0023 (14)	0.0037 (13)
C13	0.0334 (18)	0.0347 (17)	0.0415 (17)	−0.0036 (14)	0.0025 (14)	0.0021 (13)
C14	0.0335 (18)	0.0439 (19)	0.0452 (19)	−0.0010 (15)	0.0033 (15)	0.0102 (15)
C15	0.042 (2)	0.044 (2)	0.056 (2)	0.0040 (15)	0.0024 (17)	0.0040 (16)
C16	0.059 (2)	0.041 (2)	0.062 (2)	0.0030 (17)	0.0016 (19)	−0.0088 (16)
C17	0.046 (2)	0.051 (2)	0.0466 (19)	0.0015 (17)	−0.0048 (16)	−0.0036 (15)
C18	0.095 (3)	0.065 (3)	0.059 (2)	0.002 (2)	0.004 (2)	−0.0108 (19)
C19	0.040 (2)	0.063 (2)	0.071 (2)	0.0084 (17)	−0.0075 (18)	0.0232 (18)
C20	0.0361 (19)	0.056 (2)	0.0472 (18)	0.0086 (16)	−0.0053 (15)	−0.0002 (15)

### Geometric parameters (Å, °)

**Table:**

N1—C1	1.254 (4)	C9—H9A	0.9600
N1—C10	1.455 (4)	C9—H9B	0.9600
N2—C11	1.253 (4)	C9—H9C	0.9600
N2—C20	1.437 (4)	C10—C10^i^	1.517 (6)
O1—C3	1.372 (3)	C10—H10A	0.9700
O1—C8	1.428 (4)	C10—H10B	0.9700
O2—C4	1.360 (4)	C11—C12	1.456 (4)
O2—C9	1.425 (4)	C11—H11	0.9300
O3—C13	1.373 (3)	C12—C13	1.378 (4)
O3—C18	1.427 (4)	C12—C17	1.381 (4)
O4—C14	1.366 (4)	C13—C14	1.386 (4)
O4—C19	1.411 (4)	C14—C15	1.359 (4)
C1—C2	1.472 (4)	C15—C16	1.382 (5)
C1—H1	0.9300	C15—H15	0.9300
C2—C7	1.381 (4)	C16—C17	1.359 (4)
C2—C3	1.381 (4)	C16—H16	0.9300
C3—C4	1.395 (4)	C17—H17	0.9300
C4—C5	1.367 (4)	C18—H18A	0.9600
C5—C6	1.379 (4)	C18—H18B	0.9600
C5—H5	0.9300	C18—H18C	0.9600
C6—C7	1.368 (4)	C19—H19A	0.9600
C6—H6	0.9300	C19—H19B	0.9600
C7—H7	0.9300	C19—H19C	0.9600
C8—H8A	0.9600	C20—C20^ii^	1.518 (6)
C8—H8B	0.9600	C20—H20A	0.9700
C8—H8C	0.9600	C20—H20B	0.9700
			
C1—N1—C10	117.4 (3)	N1—C10—H10B	109.8
C11—N2—C20	116.3 (3)	C10^i^—C10—H10B	109.8
C3—O1—C8	114.6 (2)	H10A—C10—H10B	108.3
C4—O2—C9	117.7 (3)	N2—C11—C12	121.4 (3)
C13—O3—C18	114.0 (2)	N2—C11—H11	119.3
C14—O4—C19	116.3 (3)	C12—C11—H11	119.3
N1—C1—C2	122.3 (3)	C13—C12—C17	120.7 (3)
N1—C1—H1	118.8	C13—C12—C11	118.8 (3)
C2—C1—H1	118.8	C17—C12—C11	120.5 (3)
C7—C2—C3	118.1 (3)	O3—C13—C12	120.5 (3)
C7—C2—C1	121.9 (3)	O3—C13—C14	120.4 (3)
C3—C2—C1	119.9 (3)	C12—C13—C14	118.9 (3)
O1—C3—C2	118.4 (3)	C15—C14—O4	125.9 (3)
O1—C3—C4	121.1 (3)	C15—C14—C13	120.0 (3)
C2—C3—C4	120.4 (3)	O4—C14—C13	114.1 (3)
O2—C4—C5	123.6 (3)	C14—C15—C16	120.8 (3)
O2—C4—C3	115.8 (3)	C14—C15—H15	119.6
C5—C4—C3	120.6 (3)	C16—C15—H15	119.6
C4—C5—C6	118.7 (3)	C17—C16—C15	119.8 (3)
C4—C5—H5	120.6	C17—C16—H16	120.1
C6—C5—H5	120.6	C15—C16—H16	120.1
C7—C6—C5	120.9 (3)	C16—C17—C12	119.8 (3)
C7—C6—H6	119.5	C16—C17—H17	120.1
C5—C6—H6	119.5	C12—C17—H17	120.1
C6—C7—C2	121.2 (3)	O3—C18—H18A	109.5
C6—C7—H7	119.4	O3—C18—H18B	109.5
C2—C7—H7	119.4	H18A—C18—H18B	109.5
O1—C8—H8A	109.5	O3—C18—H18C	109.5
O1—C8—H8B	109.5	H18A—C18—H18C	109.5
H8A—C8—H8B	109.5	H18B—C18—H18C	109.5
O1—C8—H8C	109.5	O4—C19—H19A	109.5
H8A—C8—H8C	109.5	O4—C19—H19B	109.5
H8B—C8—H8C	109.5	H19A—C19—H19B	109.5
O2—C9—H9A	109.5	O4—C19—H19C	109.5
O2—C9—H9B	109.5	H19A—C19—H19C	109.5
H9A—C9—H9B	109.5	H19B—C19—H19C	109.5
O2—C9—H9C	109.5	N2—C20—C20^ii^	109.2 (3)
H9A—C9—H9C	109.5	N2—C20—H20A	109.8
H9B—C9—H9C	109.5	C20^ii^—C20—H20A	109.8
N1—C10—C10^i^	109.3 (3)	N2—C20—H20B	109.8
N1—C10—H10A	109.8	C20^ii^—C20—H20B	109.8
C10^i^—C10—H10A	109.8	H20A—C20—H20B	108.3
			
C10—N1—C1—C2	−179.3 (3)	C20—N2—C11—C12	−179.3 (3)
N1—C1—C2—C7	−13.6 (5)	N2—C11—C12—C13	168.0 (3)
N1—C1—C2—C3	167.5 (3)	N2—C11—C12—C17	−13.0 (5)
C8—O1—C3—C2	105.0 (3)	C18—O3—C13—C12	104.8 (3)
C8—O1—C3—C4	−78.0 (4)	C18—O3—C13—C14	−78.6 (4)
C7—C2—C3—O1	177.2 (3)	C17—C12—C13—O3	177.1 (3)
C1—C2—C3—O1	−3.8 (4)	C11—C12—C13—O3	−4.0 (4)
C7—C2—C3—C4	0.2 (4)	C17—C12—C13—C14	0.5 (4)
C1—C2—C3—C4	179.2 (3)	C11—C12—C13—C14	179.4 (3)
C9—O2—C4—C5	−2.7 (4)	C19—O4—C14—C15	−2.2 (4)
C9—O2—C4—C3	177.2 (3)	C19—O4—C14—C13	177.4 (3)
O1—C3—C4—O2	2.4 (4)	O3—C13—C14—C15	−177.6 (3)
C2—C3—C4—O2	179.3 (2)	C12—C13—C14—C15	−1.0 (4)
O1—C3—C4—C5	−177.7 (3)	O3—C13—C14—O4	2.8 (4)
C2—C3—C4—C5	−0.8 (4)	C12—C13—C14—O4	179.4 (2)
O2—C4—C5—C6	−179.3 (3)	O4—C14—C15—C16	−179.7 (3)
C3—C4—C5—C6	0.8 (4)	C13—C14—C15—C16	0.8 (5)
C4—C5—C6—C7	−0.2 (5)	C14—C15—C16—C17	0.0 (5)
C5—C6—C7—C2	−0.4 (5)	C15—C16—C17—C12	−0.5 (5)
C3—C2—C7—C6	0.4 (5)	C13—C12—C17—C16	0.3 (5)
C1—C2—C7—C6	−178.6 (3)	C11—C12—C17—C16	−178.6 (3)
C1—N1—C10—C10^i^	120.9 (4)	C11—N2—C20—C20^ii^	119.1 (4)

Symmetry codes: (i) −*x*+2, −*y*+2, −*z*+2; (ii) −*x*−1, −*y*+1, −*z*+1.
